# Spontaneous Intracranial Hypotension after Vestibular Schwannoma Resection Due to an Unexpected Pathology: Tarlov Cysts

**DOI:** 10.7759/cureus.1261

**Published:** 2017-05-19

**Authors:** Seth E Pross, Jeffrey D Sharon, Michael Lim, Abhay Moghekar, Aruna Rao, John P Carey

**Affiliations:** 1 Otolaryngology Head and Neck Surgery, The Johns Hopkins University School of Medicine; 2 Otolaryngology Head and Neck Surgery, University of California San Francisco; 3 Neurosurgery, The Johns Hopkins University School of Medicine; 4 Neurology, The Johns Hopkins University School of Medicine; 5 Otolaryngology Head and Neck Surgery, The Johns Hopkins University School of Medicine

**Keywords:** csf leak, tarlov cyst, vestibular schwannoma, spontaneous intracranial hypotension

## Abstract

While infrequent, cerebrospinal fluid (CSF) leaks are known to occur after surgical resection of vestibular schwannomas. Early signs of CSF leak often include headache and altered mental status. If untreated, life-threatening complications can occur, including brainstem herniation and meningitis. The appropriate surgical treatment for a CSF leak requires accurate localization of the source. While the most likely location of a CSF leak after lateral skull base surgery is through the aerated portions of the temporal bone, we present a unique case of a man with a prolonged CSF leak after an acoustic tumor removal who was ultimately found to have an occult spinal perineural (Tarlov) cyst as the source. Accurate localization was ultimately achieved with CT myelogram after empirically obliterating his mastoid failed to restore intracranial CSF volume. Tarlov cysts are the most common cause of idiopathic intracranial hypotension, and this case highlights the importance of considering this entity in the differential diagnosis of postoperative CSF leaks.

## Introduction

Cerebrospinal fluid (CSF) leaks after resection of vestibular schwannomas occur in approximately 10% of cases [1]. Signs and symptoms of CSF leak include otorrhea, rhinorrhea, positional headaches, and altered mental status. The neurotologic surgeon must be attentive to these symptoms to prevent complications, including brainstem herniation or meningitis.

While the most likely source of CSF leak after skull base surgery is in the operated area, this is not always the case. We present a unique report of a man with prolonged CSF leak after skull base surgery from an occult spinal perineural cyst to highlight this rare but important exception to the rule.   

## Case presentation

**Figure 1:**
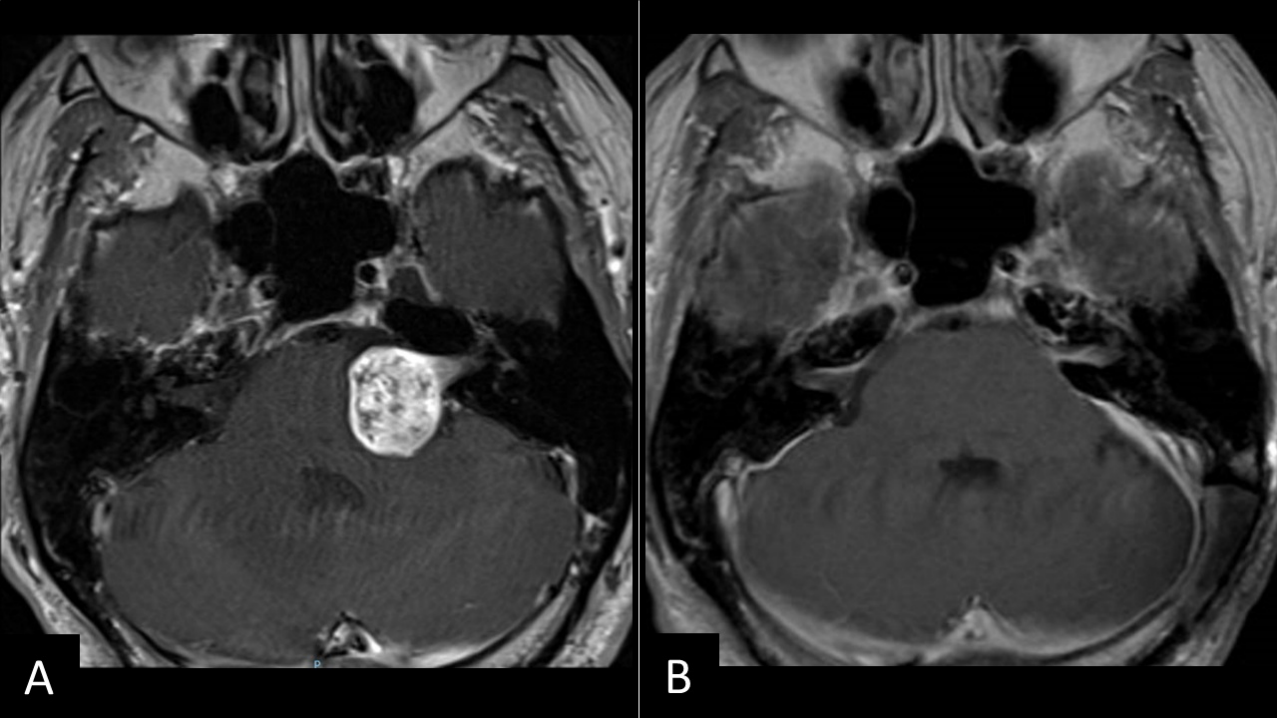
Vestibular Schwannoma Before and After Resection T1-weighted magnetic resonance imaging (MRI) demonstrating the left 2.7 cm vestibular schwannoma (A) before and (B) nine months after surgical resection and reconstruction of the internal auditory canal (IAC) bone with hydroxylapatite.

A 56-year-old man underwent suboccipital surgical resection of a 2.7 cm vestibular schwannoma without a lumbar drain (Figure 1). In the early postoperative period, he had no signs or symptoms of CSF leakage. Nine months later, he developed vomiting, fatigue, poor mentation, and headaches that worsened with coughing and straining. He reported occasional small volume rhinorrhea, which was not associated with straining or head position. On exam, the middle ear was aerated and rhinorrhea could not be provoked with dependent positioning.

**Figure 2:**
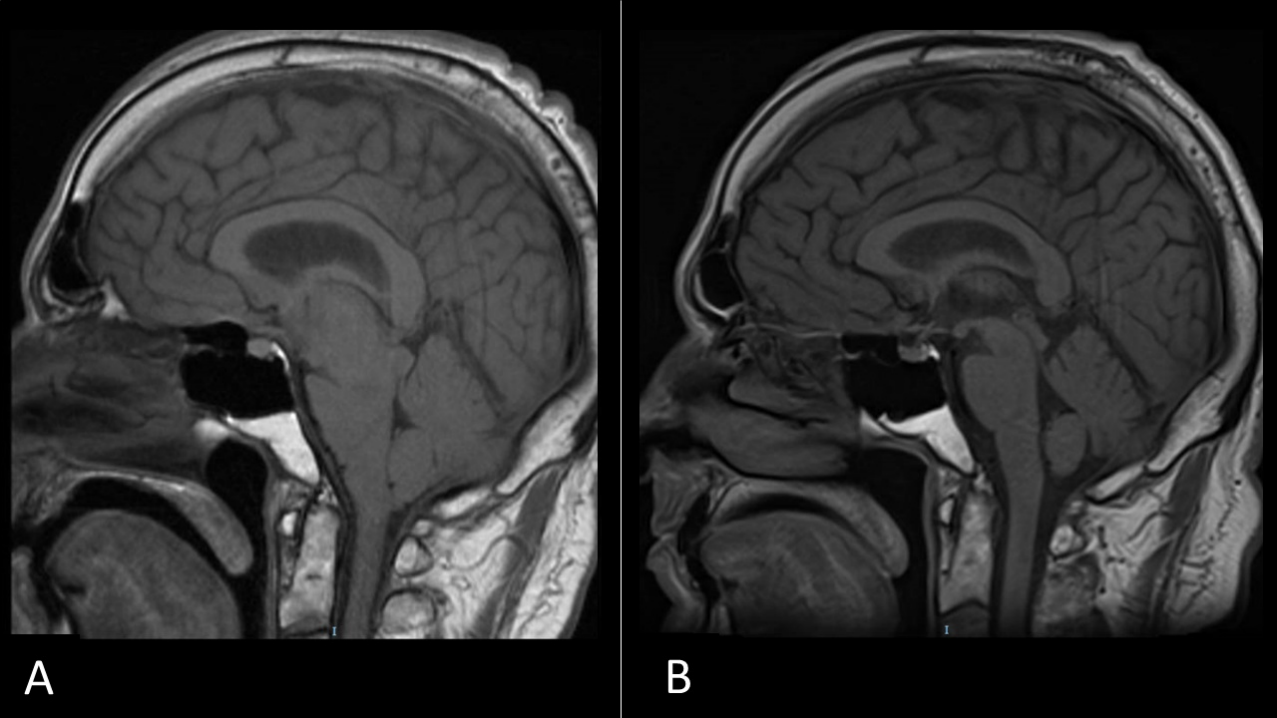
Intracranial Hypotension Before and After Dural Repair T1-weighted sagittal MRI nine months after surgical resection (A) showing brain stem sagging, flattening of the pons, decreased pontomesencephalic angle, decreased mamillopontine distance, and mild uncal herniation consistent with intracranial hypotension and (B) resolution of these findings six weeks after blood and fibrin glue patching.

**Figure 3:**
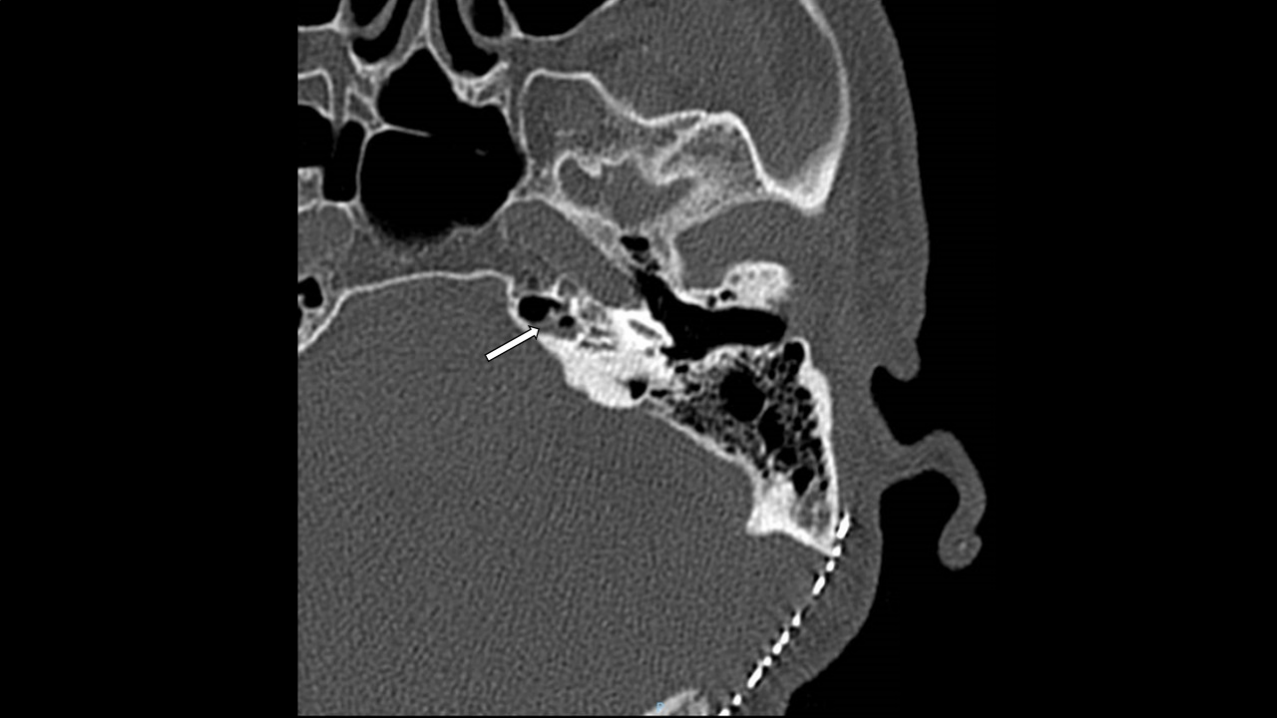
CT Temporal Bone CT temporal bone nine months after tumor resection demonstrating small air-fluid level (white arrow) in the petrous apex air cells adjacent to the petrous carotid and internal auditory canal.

Brain magnetic resonance imaging (MRI) with contrast showed signs of significant intracranial hypotension, including brain stem sagging, flattening of the pons, diffuse dural thickening, enhancement and partial effacement of the basal cisterns, and mild uncal herniation (Figure 2A). A temporal bone computed tomography (CT) scan demonstrated a small air-fluid level in the air cells adjacent to the internal auditory canal, suggesting a CSF leak through the temporal bone (Figure 3).

A presumed diagnosis of iatrogenic CSF otorhinorrhea was treated with Eustachian tube plugging and mastoid obliteration with abdominal fat graft. However, no definitive source of CSF was identified intraoperatively. Symptoms initially improved; however, one month later his headaches returned, and a repeat MRI was unchanged.

**Figure 4:**
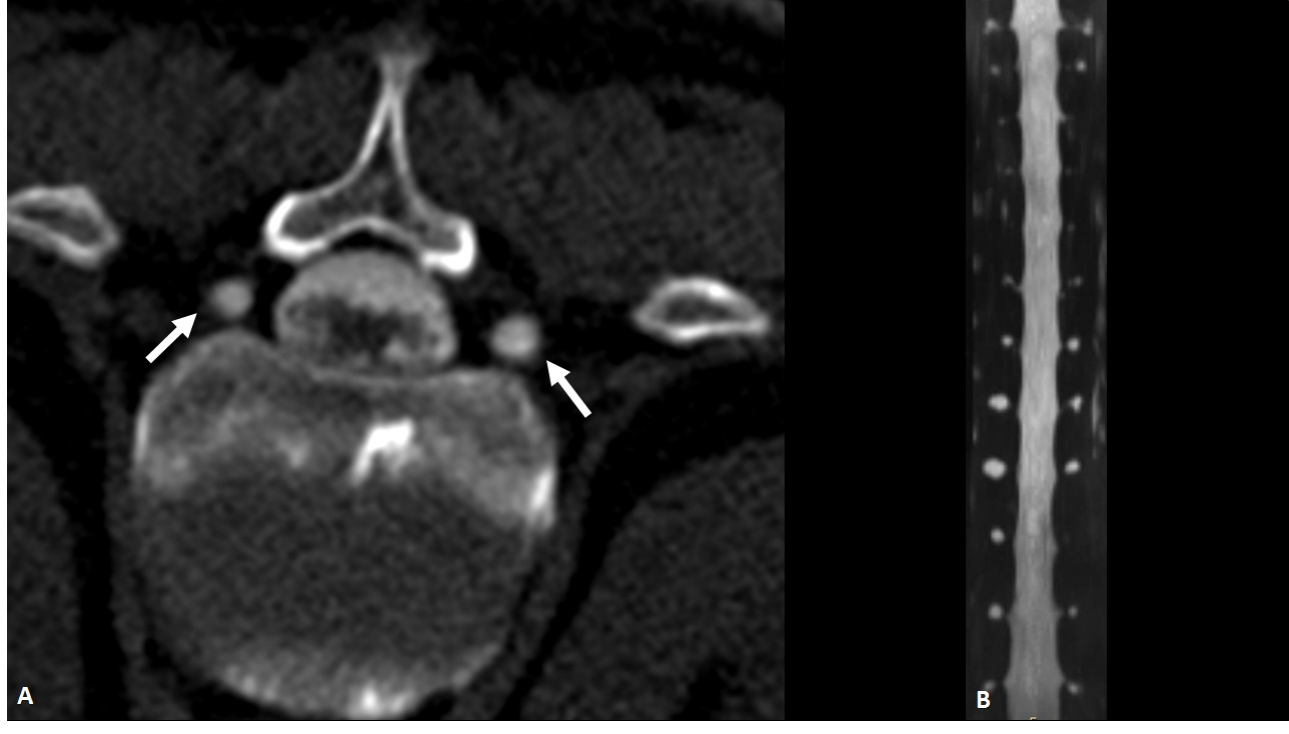
CT Myelogram CT myelogram in (A) axial and (B) coronal reconstruction demonstrating bilateral small perineural (Tarlov) cysts of the spine at multiple levels (white arrows).

Given the concern for an occult extracranial CSF leak, imaging of the spine was performed. While the MRI did not show evidence of spinal CSF extravasation, a CT myelogram revealed multiple small perineural cysts of the thoracic spine (Figure 4). These were empirically treated with an autologous blood patch with fibrin glue followed by a surgical duraplasty of four thoracic cysts, which resolved his symptoms and his MRI findings (Figure 2B). 

## Discussion

Initially described by Tarlov in 1935, perineural (Tarlov) cysts are CSF-filled cysts that arise at the junction of the dorsal ganglion and nerve root [2]. Most commonly found in the sacral region, they can occur anywhere along the spine. Typically, these lesions are asymptomatic but can cause back pain, radicular symptoms, and CSF leaks. Tarlov cysts have been treated via surgical excision, blood patching, and percutaneous drainage [3-4].

In our patient, several factors led us to erroneously conclude that the CSF leak was at the surgical site. These factors included the timing of the leak after surgery, the self-reported rhinorrhea, and ambiguous temporal bone CT findings. Furthermore, the patient did not endorse several characteristic symptoms of Tarlov cysts, including radicular pain and bowel or bladder dysfunction. However, his symptoms of a positional headache, fatigue, and decreased mental clarity recurred after standard surgical obliteration of the mastoid and plugging of the Eustachian tube, which led us to suspect an extracranial site of CSF loss. Ultimately, the CT myelogram was diagnostic.

Tarlov cysts are the main cause of spontaneous intracranial hypotension. Given the onset of symptoms several months after surgery, we hypothesize that this case was caused by a spontaneous cyst rupture, unrelated to our operation. However, it is possible that the cyst ruptured perioperatively due to changes in CSF pressure from CSF loss, positioning, and intracranial irrigation, as has been reported for subarachnoid hemorrhage surgery [5]. Additionally, our patient had a Marfanoid body habitus, which may be a risk factor [6].

## Conclusions

When CSF leaks occur after skull base surgery, they are typically treated with either conservative measures, surgical repair of the operated site, or obliteration of downstream CSF pathways. However, CSF leaks can occur at distant sites, including anywhere along the spinal column. We present a rare case of an occult CSF leak from perineural cysts which occurred after surgical resection of a vestibular schwannoma. The source of the leak was identified only after failure of mastoid obliteration with Eustachian tube plugging led to consideration of a remote source of the leak. While rare, perineural (Tarlov) cysts are the most common cause of spontaneous intracranial hypotension, and they should be considered in the differential diagnosis of postoperative CSF leaks. 

## References

[1] Selesnick SH, Liu JC, Jen A, Newman J (2004). The incidence of cerebrospinal fluid leak after vestibular schwannoma surgery. Otol Neurotol.

[2] Tarlov I (1938). Perineural cysts of the spinal nerve roots. Arch Neur Psych.

[3] Voyadzis JM, Bhargava P, Henderson FC (2001). Tarlov cysts: a study of 10 cases with review of the literature. J Neurosurg.

[4] Murphy K, Oaklander AL, Elias G, Kathuria S, Long DM (2016). Treatment of 213 patients with symptomatic Tarlov cysts by CT-guided percutaneous injection of fibrin sealant. AJNR Am J Neuroradiol.

[5] Sivakumar W, Ravindra VM, Cutler A, Couldwell WT (2014). Intracranial hypotension in the setting of concurrent perineural cyst rupture and subarachnoid hemorrhage. J Clin Neurosci.

[6] Wang B, Moon SJ, Olivero WC, Wang H (2014). Pelvic pain from a giant presacral Tarlov cyst successfully obliterated using aneurysm clips in a patient with Marfan syndrome. J Neurosurg Spine.

